# COVID-19 백신접종 시작 단계에서 간호사의 백신접종 수용도와 관련 요인

**DOI:** 10.7586/jkbns.24.013

**Published:** 2024-08-23

**Authors:** Kyoung Ha Kim, Jae Sim Jeong

**Affiliations:** 1 Department of Nursing. Asan Medical Center, Seoul, Korea; 1 서울아산병원 간호부; 2 Department of Clinical Nursing, University of Ulsan, Ulsan, Korea; 2 울산대학교 산업대학원 임상전문간호학

**Keywords:** COVID-19 vaccines, Nurses, Vaccination, Acceptance, COVID-19 백신, 간호사, 백신접종, 수용도

## Abstract

**Purpose:**

This study aimed to identify factors associated with nurses' vaccination acceptance in the initial stage of coronavirus infectious disease 2019 (COVID-19) vaccination in Korea.

**Methods:**

This cross-sectional study collected data from hospital-level or higher medical institutions from May 5 to May 7, 2021 with a total of 368 nurses via an online survey. Multiple regression analysis was used to identify factors associated with vaccination acceptance.

**Results:**

The acceptance of COVID-19 vaccination had an average score of 4.28 ± 0.80 out of 7 points. Among the sub-areas of acceptance, the safety of the vaccine exhibited the lowest score (average, 2.80 ± 0.83). The factors related to COVID-19 vaccination acceptance in nurses were age (over 40 compared to under 29) (β = .16, *p* = .008), experience (more than 11 years compared to under 5) (β = .16, *p* = .010), the absence of a preferred vaccine (β = -.14, *p* = .004), and educational background (a master's degree or higher compared to a bachelor's degree or lower) (β = .12, *p* = .042).

**Conclusion:**

In the initial stage of COVID-19 vaccination, nurses believed that the COVID-19 vaccine was important and that people should be vaccinated, but had concerns regarding the safety of the vaccine. Older age, more experience, the absence of a preferred vaccine, and a higher educational background were associated with higher acceptance of COVID-19 vaccination. Therefore, more active education on the necessity and value of vaccination for young people is required at the individual and socio-cultural level, and a sufficient supply of preferred vaccines at the national level is needed.

## 서론

### 1. 연구의 필요성

2019년 12월 중국 우한에서 시작된 코로나바이러스감염증-19 (coronavirus infectious disease 2019, COVID-19)가 전 세계로 확산되어, 20년 1월 5일부터 24년 5월 19일까지 WHO 웹사이트에서 수집한 COVID-19의 전 세계 유병률 데이터에 따르면 775,522,404명의 확진자와 7,049,617명의 사망자가 발생했다[1].

백신이 COVID-19를 예방하기 위해서는 인구의 75%가 백신접종을 한다는 전제 하에 최소 70%의 효능을, 사회적 거리두기 등 다른 조치 없이 COVID-19를 진압하기 위해서는 최소 80%의 효능을 가져야 한다고 알려져 있다[2]. 이에 전 세계가 집단 면역 형성을 위해 COVID-19 백신접종에 박차를 가하여 2020년 12월 14일 COVID-19 백신 제품 최초 출시일을 기준으로 총 COVID-19 백신 접종량은 54억 7천만 건에 달하였다[3].

국내 첫 COVID-19 백신접종은 2021년 2월 26일부터 시작하였으며, 대상은 65세 미만 요양기관 입소자 및 종사자와 의료기관 종사자 등 크게 4분류로 나누어 우선순위에 따라 시행되었다. 특히 COVID-19 관련 업무에 종사하거나 환자치료에 참여가능성이 있는 의료종사자가 우선순위로 선정되어 2021년 3월 4일부터 접종을 시작하였다[4]. 따라서 백신접종이 시작되었을 당시 의료종사자 본인의 접종의사결정을 할 수 있는 시간이 짧았고, 사회적인 분위기 또한 접종에 대한 자율성을 존중하기 보다는 적극적인 접종권고로 이어졌다[5].

그리고 COVID-19 백신은 개발과정이 짧고 새로운 방법으로 제작되어 안전성 부분에 있어 정보가 제한적이었는데, 정확한 정보를 인식하기도 전에 백신접종이 진행되었다[5]. 잘못된 정보가 백신에 대한 대중의 불안을 증가시키고 백신 정책을 정치화할 수 있는 상황에서 백신접종 수용을 정확하게 측정하는 것은 중요하다[6]. 의료종사자 중 가장 많은 인원을 차지하는 간호사는 신뢰할 수 있는 백신 정보 관련 출처로서 백신접종 혜택에 대한 메시지를 전달하고 새로 개발된 백신에 대한 환자의 걱정과 우려를 완화시킬 수 있다[7]. 또한 간호사는 COVID-19 환자에 쉽게 노출될 가능성이 높은 최전선 의료제공자이면서, 동시에 병원 내 감염 확산에 중요한 역할을 할 수 있다.

국외의 COVID-19 백신접종 수용도에 관한 연구는 이미 2020년 초반부터 대규모의 인구집단을 대상으로 진행되었고[8-12], 국내에서는 백신접종 전인 2021년 2월 중순 간호사를 대상으로 수용도를 조사한 Park과 Ha[13]의 연구가 있었다. 선행연구[8-15]에서 COVID-19 백신접종 수용도와 관련된 요인으로 성별, 연령, 인종, 병력 등 인구사회학적 특성 뿐만 아니라 COVID-19 관련 경험적 특성 및 백신접종 관련 특성, 정책과 관련된 여러 요인들이 복합적으로 작용하는 것으로 나타나, 이를 규명하기 위한 반복 연구가 필요하였다.

이에 본 연구는 2021년 5월 초 국내의 COVID-19 백신접종이 이미 시작되고 있는 상태였지만 의무적 접종이라는 사회적인 분위기를 고려하여 간호사의 백신접종 수용도를 파악하고 관련 요인을 분석하여 의료종사자 뿐만 아니라 범국민 COVID-19 백신접종 수용도를 높이기 위한 근거를 제공하고자 하였다.

### 2. 연구의 목적

본 연구의 목적은 간호사의 COVID-19 백신접종 수용도와 관련 요인을 분석하기 위함이며, 이를 위한 구체적인 목적은 다음과 같다.

첫째, 대상자의 COVID-19 백신접종 수용도를 확인한다.

둘째, 대상자의 일반적 특성과 일반적 특성에 따른 백신 접종 수용도의 차이를 파악한다.

셋째, COVID-19 백신접종 수용도와 관련된 요인을 규명한다.

## 연구 방법

### 1. 연구 설계

병원급 이상 의료기관에 종사하는 간호사를 대상으로 COVID-19 백신접종 시작 단계에서 백신접종 수용도와 관련 요인을 분석하기 위한 단면 조사 연구이다.

### 2. 연구 대상

본 연구의 표적 모집단은 의료법 제3조[16]에 정의되어 있는 병원급 이상 의료기관(병원, 치과병원, 한방병원, 요양병원, 정신병원, 종합병원, 상급종합병원)에서 근무하는 간호사이다. COVID-19 백신접종 유무와 상관없이 대상자가 될 수 있으나, 당시 백신 개발과정에서 임상시험에 포함되지 않아 접종대상에 해당되지 않는 임신부는 제외하였다. 본 연구의 표본은 인터넷 포털 사이트(너스케입 https://www.nurscape.net)와 온라인 커뮤니티(간호사에 대한 모든 것 https://cafe.naver.com/angel2nurse)를 방문하는 간호사 중 본 연구에 자발적으로 참여하기로 동의한 자로 하였다. 대상자 수는 G*Power ver. 3.1.9.7. 프로그램을 사용하여 계산하였으며, 다중 회귀분석을 기준으로 효과크기(effect size)= .15, 유의수준(α)= .05, 검정력(1-β)= .95, 변수 34개로 산출하였을 때 필요한 표본 수는 최소 274명이었다[17]. 탈락률 10.0%를 고려하여 305명을 대상자로 선정하였으나, 총 372명이 설문에 응답하였다. 그 중 본 연구 대상자에 해당되지 않는 설문 4건을 제외하여 최종 368건의 자료를 분석하였다.

### 3. 연구 도구

#### 1) COVID-19 백신접종 수용도

COVID-19 백신접종 수용도는 아동용 백신에 대한 백신접종 수용도를 측정하기 위해 Sarathchandra 등[6]이 개발한 도구를 연구자가 저자의 승인을 받아 COVID-19에 맞게 수정, 보완하였다. 원 도구는 5개 하위영역의 총 20개의 문항으로 구성된다. 원 도구의 문항 중 아동을 대상으로 한 내용을 의료종사자 대상으로 변경하였으며, 아동용 백신을 COVID-19 백신으로 변경하였다. 또한 아동용 백신의 성분과 관련된 문항을 제외하였으며, 국내에서 백신접종 우선순위로 정해진 노인과 만성질환자의 백신 일정에 대한 문항을 추가하였다. 본 연구자에 의해 수정, 보완한 측정도구는 전문가 그룹(감염관리 전공 간호학 교수 2명, 감염내과 전문의 1명, 감염관리 전문간호사 2명)에게 총 3회의 내용타당도 검증을 실시하여, 전문가의 의견을 토대로 문항 내용과 어휘를 수정, 보완하였으며 최종적으로 5개 하위영역의 총 19개의 문항으로 구성하였다. 각 하위영역은 백신의 이상반응과 관련하여 안전성을 다루는 백신에 대한 인지된 안전성, 백신의 효능에 대한 인식을 다루는 백신에 대한 인지된 효과와 필요성, 백신의 접종 순위와 일정에 대한 우려에 초점을 맞춘 백신의 선택 및 일정, 백신 수용 또는 주저로 기울일 수 있는 가치 기반 감정을 다루는 백신에 대한 긍정적인 가치와 영향, 백신접종에 대한 공공 정책과 관련된 신념을 확인하는 백신접종을 요구하는 국가의 정당성 인지로 모든 문항은 내용타당도 지수(content validity index) 0.8~1.0으로 확인되었다. 하위영역에 따른 각 문항은 ‘전혀 그렇지 않다’ 1점에서 ‘매우 그렇다’ 7점까지의 7점 척도로, 이중 부정 문항은 역환산 처리하였다. Sarathchandra 등[6]의 연구에서 개발 당시 도구의 신뢰도 Cronbach’s α는 .97이었으며 본 연구의 신뢰도 Cronbach’s α는 .86이었다.

#### 2) 대상자 특성

인구사회학적 특성과 근무지 관련 특성, COVID-19 관련 경험적 특성, 백신접종 관련 특성으로 구성된 연구자가 개발한 도구를 사용하였다. 인구사회학적 특성은 성별, 연령, 결혼상태, 교육 정도, 만성질환 여부(예/아니오)로 구성하였으며, 근무지 관련 특성은 총 임상근무 경력, 현재 근무 병원의 종류 및 병상 수, 현재 근무 부서로 구성하였다. 이 중 현재 근무부서는 COVID-19 세계적 대유행인 상황에 맞추어 병동, 중환자실, 응급실, 확진자 치료 부서로 구성된 COVID-19 고위험 부서와 외래, 수술실, 검사실, 상근 부서 등으로 구성된 COVID-19 저위험 부서로 분류하였다. COVID-19 관련 경험적 특성은 COVID-19 확진자 및 국가기준 접촉자(자가격리대상자, 능동감시대상자, 보건교육대상자) 경험 여부(예/아니오), 간호 경험 여부(예/아니오)로 구성하였으며, 백신접종 관련 특성은 이전 독감 시즌 독감 백신접종 여부(예/아니오), 선호하는 COVID-19 백신, COVID-19 백신접종 여부(예/아니오)와 그에 따른 부가적인 사항(사전 정보 획득과 접종 의향)으로 구성하였다.

### 4. 자료 수집 및 윤리적 고려

자료수집 전 연구자가 소속된 서울아산병원 연구윤리심의위원회(IRB)의 심의와 승인(승인번호: 2021-0620)을 받고 자료를 수집하였다. 자료 수집기간은 2021년 5월 5일부터 5월 7일까지였다. 간호사들이 소통하는 공간인 인터넷 포털 사이트(너스케입 https://www.nurscape.net)와 커뮤니티(간호사에 대한 모든 것 https://cafe.naver.com/angel2nurse)의 게시판을 이용하여 연구목적과 방법, 대상자 선정기준이 기재된 모집 공고문과 더불어 온라인 설문지를 배포하였다. 온라인 설문지(https://forms.gle/sgLXrM6ixC2TepNe9)에는 자발적으로 참여를 원하는 대상자에게 연구를 시행하며, 수집된 자료는 연구 이외의 목적으로 사용되지 않을 것이며 대상자가 원하면 언제든지 철회할 수 있고, 비밀유지, 익명성 보장 등에 대해 기술되어 있는 설명문을 함께 포함하였다. 연구자료와 관련된 모든 파일은 암호화하여 연구자 외에는 접근할 수 없도록 개인 노트북에 보관하였으며, 연구자에 의해서만 자료가 다루어지도록 하였다.

### 5. 자료 분석

수집된 자료는 SPSS 프로그램(IBM SPSS Statistics for Windows, Version 24.0. Armonk, NY, US)을 이용하여 분석하였다. 대상자의 COVID-19 백신접종 수용도와 대상자 특성은 실수와 백분율 또는 평균과 표준편차로 분석하였고, Shapiro-Wilk 방법을 이용하여 정규성을 검정하였다. 대상자 특성에 따른 COVID-19 백신 수용도의 차이는 independent t-test나 one-way ANOVA로 분석하고 사후검정은 Scheffé test를 사용하였다. 단, 등분산성이 성립하지 않은 경우 Welch’s ANOVA로 분석하였다. COVID-19 백신접종 수용도와 관련된 요인을 파악하기 위해 다중회귀분석(multiple regression) 방법 중 단계적 변수 선택방법으로 분석하였는데, 분석에 투입된 독립변수는 일반적 특성에 따른 COVID-19 백신접종 수행도에 유의한 차이를 나타낸 나이, 결혼 여부, 학력, 총 근무 경력, 근무 부서, 선호 백신 여부이다. 모든 경우의 통계적 유의수준은 *p* < .05를 기준으로 하였다.

## 연구 결과

### 1. COVID-19 백신접종 수용도

COVID-19 백신접종 수용도는 7점 만점에 평균 4.28 ± 0.80점이며 하위영역으로 백신에 대한 인지된 안전성(항목 1-4)은 2.80 ± 0.83점, 백신에 대한 인지된 효과 및 필요성(항목 5-8) 4.63 ± 0.86점, 백신의 선택 및 일정(항목 9-12) 4.85 ± 1.24점, 백신에 대한 긍정적인 가치와 영향(항목 13-16) 4.97 ± 1.21점, 백신접종을 요구하는 국가의 정당성 인지(항목 17-19) 4.10 ± 1.02점이었다. 문항별 COVID-19 백신접종 수용도에서 백신접종 수용도가 높은 문항은 10번 ‘의료종사자에게 COVID-19 백신을 우선 접종하는 것은 타당하다(평균 5.62 ± 1.50점)’, 19번 ‘의료종사자는 정부의 COVID-19 백신접종 지침을 준수해야 한다(평균 5.52 ± 1.37점)’, 14번 ‘COVID-19 백신접종으로 환자와 다른 의료종사자를 COVID-19 감염으로부터 보호할 수 있다(평균 5.43 ± 1.36점)’ 순이었다. 반면 백신접종 수용도가 낮은 문항은 3번 ‘COVID-19 백신은 경미한 이상반응(예: 근육통, 발열 등)을 유발할 수 있다(평균 1.56 ± 1.12점)’, 18번 ‘의료종사자의 COVID-19 백신접종은 자발적이어야 한다(평균 2.13 ± 1.30점)’, 2번 ‘COVID-19 백신은 심각한 이상반응(예: 아나필락시스 반응)을 유발할 수 있다(평균 2.58 ± 1.27점)’이었다(Table 1).

### 2. 대상자 특성 및 대상자 특성에 따른 COVID-19 백신접종 수용도

#### 1) 인구사회학적 특성과 근무지 관련 특성

총 대상자는 368명으로 성별은 여자가 362명(98.4%), 평균 연령은 31.92세로 29세 이하가 167명(45.3%)으로 가장 많았다. 결혼 여부는 미혼이 223명(60.6%), 학력은 학사학위 이하가 270명(73.4%)이었다. 만성 질환은 27명(7.3%)이 있다고 하였으며, 총 경력은 평균 8.27 ± 6.71년으로 5년 이하가 143명(38.9%)으로 가장 많았고, 현재 근무 병원은 상급종합병원 265명(72.0%), 현재 근무하고 있는 병원의 병상 수는 1,000병상 이상이 214명(58.1%)으로 가장 많았다. 근무 부서는 일반 병동 161명(43.8%), 중환자실 62명(16.8%), 응급실 29명(7.9%), COVID-19 치료 부서 6명(1.6%)로 구성된 COVID-19 고위험 부서가 258명(70.1%)이었으며, 외래 32명(8.7%), 수술실 19명(5.2%), 이 외에 검사실, 상근 부서 등 59명(16.0%)으로 구성된 COVID-19 저위험 부서가 110명(29.9%)이었다. 인구사회학적 특성과 근무지 관련 특성 중 COVID-19 백신접종 수용도에 유의한 변수는 나이, 결혼 여부, 학력, 총 근무 경력, 근무 부서였다. 대상자의 나이에 따라 29세 이하에 비해 30~39세가, 30~39세에 비해 40세 이상에서 수용도가 높았으며(F = 24.98, *p* < .001), 미혼인 대상자에 비해 기혼인 대상자가 백신접종 수용도(t = −4.13, *p* < .001)가 높았다. 석사학위 이상 소지자가 학사학위 이하 소지자에 비해 수용도가 높았으며(t = −5.33, *p* < .001), 총 근무 경력은 5년 이하, 6~10년에 비해 11년 이상이 유의하게 높았다(F = 20.38, *p* < .001). 근무 부서는 COVID-19 고위험 부서에 비해 저위험 부서에서 수용도가 높았다(t = 2.88, *p* = .004). 반면, 성별, 만성 질환 여부, 현 근무 병원 및 병원의 병상 수는 COVID-19 백신접종 수용도에 유의한 차이가 없었다(Table 2).

#### 2) COVID-19 관련 경험적 특성

최근 일 년 이내 COVID-19에 노출된 경험이 있는 대상자는 94명(25.5%)이었고, 확진자 7명(7.4%), 자가격리대상자 34명(36.2%), 능동감시대상자 33명(35.1%), 보건교육대상자 43명(45.7%)이었다. COVID-19 확진자 및 국가기준 접촉자를 간호한 경험이 있는 대상자는 132명(35.9%)으로 그 중 확진자 60명(45.5%), 국가기준 접촉자인 자가격리대상자 61명(46.2%), 능동감시대상자 39명(29.5%), 보건교육대상자 42명(31.8%)을 간호한 경험이 있다고 응답하였다. COVID-19 관련 경험적 특성 중 COVID-19 백신접종 수용도에 유의한 차이가 있는 변수는 없었다(Table 3).

#### 3) 백신접종 관련 특성

2020년 가을에 인플루엔자 백신접종을 한 대상자는 329명(92.1%)이었다. 330명(89.7%)이 COVID-19 백신접종을 완료하였고 이 중 Astrazeneca 백신 1차 접종 282명(85.5%), Astrazeneca 백신 2차 접종 1명(0.3%), Pfizer 백신 1차 접종 2명(0.6%), Pfizer 백신 2차 접종 29명(8.8%)으로 응답자의 많은 수가 Astrazeneca 백신을 접종하였다. 선호하는 COVID-19 백신이 있다고 응답한 대상자는 226명(61.4%)이었으며, 선호하는 백신은 Pfizer 백신이 203명(89.4%), 그 외 23명(10.6%)이었다. COVID-19 백신접종을 받은 330명을 대상으로 접종 전 해당 백신에 대해 어떤 정보를 받았는지에 대해서는 접종 방법 271명(82.1%), 접종 간격 284명(86.1%), 효과 229명(69.4%), 이상반응 299명(90.6%), 이상반응 시 대처방법 276명(83.6%), 접종 후 주의사항 274명(83.0%)으로 응답하였다. 가족이나 지인에게 COVID-19 백신접종에 대해 ‘백신접종을 강하게 권고할 것이다’라고 응답한 대상자는 25명(7.6%)이었으며, ‘정해진 시기와 순서에 맞게 접종을 권유할 것이다’에 197명(59.6%), ‘백신접종 연기를 권유할 것이다’에 69명(20.9%), ‘백신접종을 하지 말라고 권유할 것이다’에 22명(6.7%)이 응답하였다. 반면, 백신접종을 하지 않은 대상자 38명 중 앞으로의 접종 계획에 대해서 ‘COVID-19 백신 종류에 상관없이 접종기회가 된다면 접종을 받을 것이다’라고 응답한 대상자는 9명(23.7%)이었으며, ‘COVID-19 백신 종류에 따라 접종시기를 조절할 것이다’에 21명(55.2%), ‘COVID-19 백신 종류에 상관없이 접종 받지 않을 것이다’에 8명(21.1%)이 응답하였다. 백신접종 관련 특성 중 선호 백신 여부, 백신접종 전 받은 정보, 백신접종 후 추천 여부, 접종 받지 않았을 경우 미래 접종계획에서 유의한 차이가 있었다. 선호하는 COVID-19 백신이 없는 경우 백신접종 수용도가 높았으며(t = −3.49, *p* = .001), 백신접종 전 해당 백신에 대해 효과(t = 2.41, *p* = .017), 이상반응(t = 2.54, *p* = .019), 이상반응 시 대처방법(t = 2.77, *p* = .006), 접종 후 주의사항(t = 4.49, *p* < .001)에 대한 정보를 받을수록 백신접종 수용도가 높았다. 백신접종 후 추천 여부에서는 ‘백신접종을 강하게 권고할 것이다’, ‘정해진 시기와 순서에 맞게 접종을 권유할 것이다’, ‘백신접종 연기를 권유할 것이다’, ‘백신접종을 하지 말라고 권유할 것이다’ 순으로 유의하게 높았다(F = 88.70, *p* < .001). 백신접종을 받지 않은 응답자를 대상으로 앞으로의 접종계획에서는 ‘COVID-19 백신 종류에 상관없이 접종기회가 된다면 접종을 받을 것이다’가 ‘COVID-19 백신 종류에 따라 접종시기를 조절할 것이다’, ‘COVID-19 백신 종류에 상관없이 접종 받지 않을 것이다’에 비해 유의하게 높았다(F = 9.58, *p* < .001). 반면, 인플루엔자 백신접종 여부, COVID-19 백신접종 여부는 COVID-19 백신접종 수용도에 유의한 차이가 없었다(Table 4).

### 3. COVID-19 백신접종 수용도 관련 요인

COVID-19 백신접종 수용도에 유의하게 차이를 보였던 나이, 결혼 여부, 학력, 총 근무 경력, 근무 부서, 선호 백신 여부를 독립변수로 다중회귀분석을 실시하였다. 단, 접종 후 추천 여부 및 접종 받지 않았다면 앞으로의 접종계획은 COVID-19 백신접종 수용도에 유의한 차이를 보였으나, 백신접종 의도 자체를 묻는 문항이므로 다중회귀분석에서 제외하였다. 나이와 총 근무 경력은 가변수(dummy variable)로 전환하였고, Durbin-Watson 지수는 2.08으로 2에 근사한 값을 보여 자기상관의 문제는 없었다. 분산팽창지수(variance inflation factor)는 1.02-1.64로 다중공선성이 없었으며 회귀분석을 실시하기에 적합하였다(F = 16.83, *p* < .001). 선형회귀분석을 실시한 결과 모형의 설명력은 14.7%이었다. 간호사의 COVID-19 백신접종 관련 요인은 나이(29세 이하에 비해 40세 이상) (β = .16, *p* = .008), 경력(5년 이하에 비해 11년 이상) (β = .16, *p* = .010), 선호하는 백신이 없는 경우(β = -.14, *p* = .004), 학력(학사학위 이하 소지자에 비해 석사학위 이상 소지자) (β = .12, *p* = .042)이었다(Table 5).

## 논의

본 연구는 국내에서 COVID-19 백신이 처음 도입되고, 국가 정책 및 우선순위에 따라 의료종사자를 대상으로 백신접종이 이루어지는 시작 단계에서 병원급 이상 의료기관에 종사하는 간호사를 대상으로 COVID-19 백신접종 수용도 수준과 수용도와 관련된 요인을 파악하기 위한 조사연구이다.

본 연구의 COVID-19 백신접종 수용도는 61.1%로, 본 연구와 다른 도구를 사용하여 일반인을 대상으로 한 연구에서의 수용도 67%~91%[9,11,12,15]보다는 낮았지만, 의료종사자를 대상으로 한 9개 연구의 체계적 문헌고찰을 시행한 Luo 등[18]의 연구에서 평균값 51%보다는 높았다. 하지만, 국내 간호사를 대상으로 2월에 시행한 Park과 Ha[13]의 연구에서 74.5%였던 것에 비해 낮은 수치를 보여, 백신접종이 시작된 이후 설문조사를 시행할 당시 Astrazeneca 백신과 혈소판 감소를 동반한 혈전증 간의 인과성을 비롯한 백신 안전성 논란과 관련하여 감소한 경향을 보인 것이라 추측된다. 단, 본 연구는 백신접종 중에 설문조사를 시행한 연구로 앞서 언급된 백신접종 전 설문조사를 시행한 연구와는 조사 시점이 다르기에 직접적으로 비교하기가 어려울 수 있다.

COVID-19 백신접종 수용도 각 하위 영역을 살펴보면 안전성과 관련된 항목에서 평균 점수가 가장 낮았으며, 그 중에서도 이상반응과 관련된 문항이 가장 낮았다. 이는 본 연구 대상자의 89.7%가 이미 백신접종을 했음을 고려할 때, 접종을 받은 상당수가 이상반응을 겪었으리라 생각되며, 질병관리청에서 발표한 정례브리핑 자료에서도 2021년 10월 31일 기준 전체 백신접종 78,392,936건 중 이상반응 신고는 353,535건으로 신고 사례 중 근육통, 두통, 발열 등 일반 이상반응이 96.4%(340,715건)이었음을 확인할 수 있었다[19]. 또한 2021년 5월 말 의료종사자를 대상으로 백신접종 영향요인과 접종 후 이상 반응 실태조사를 시행한 Lee와 Choi[5]의 연구에서도 전체 접종인원의 70.9%가 이상반응이 있었다고 보고하였다. 따라서 백신의 이상반응에 대해 정확하고 투명하게 정보를 제공하고, 신뢰할 수 있는 이상반응 감시 체계를 운영하여 의료종사자 뿐만 아니라 범국민이 안심하고 접종 받을 수 있도록 해야 할 것이다.

그리고 의료종사자의 백신접종 자발성과 관련된 문항이 이상반응 다음으로 평균 점수가 낮았는데, 이는 대상자의 대다수가 이미 백신접종을 시행하였음을 고려했을 때 초기 의료종사자를 대상으로 진행된 백신접종이 자발적이기 보다는 의무적으로 진행되었기 때문인 것으로 추측할 수 있다. 의무적인 인플루엔자 백신접종에 대한 의료종사자의 태도를 분석한 Gualano 등[20]의 연구에서 의료종사자의 61%가 필수 인플루엔자 백신접종에 동의한 것에 비하면 본 연구 문항의 수용도는 30.4%로 낮은 수준이었다. 하지만 ‘정부가 의료종사자에게 COVID-19 백신접종을 요구하는 것은 정당하다’, ‘의료종사자는 정부의 COVID-19 백신접종 지침을 준수해야 한다’ 문항에는 각각 전체 수용도의 평균보다 점수가 높았기에 국가 정책에 따라 의무 백신접종이 필요함에 대해서는 인지하고 있음을 알 수 있다. 이는 Park과 Ha[13]의 연구에서 접종의도에 전문직 의무감의 영향이 가장 큰 것으로 확인된 것과 유사하다. 하지만 전문직 의무감과 강제적인 접종에 대한 개인의 견해 사이에서 이중적인 감정을 가지고 있다고 추측되므로 전문직 의무감을 고취시키면서 의무적인 정책이 효과적일 수 있도록 인적, 물적 자원 등 국가의 제도적인 지원이 요구된다.

COVID-19 백신접종 수용도와 관련된 요인은 나이, 경력, 선호 백신 여부, 학력이었다. 연구 대상자의 평균 나이는 31.92세로 29세 이하에 비해 40세 이상이 백신접종 수용도가 높았다. 연령대가 높을수록 수용도가 높은 것은 선행 연구 결과[5,9,11,14,15,18,21]와 일치한다. 연구 대상자의 평균 경력은 8.27년으로 5년 이하에 비해 11년 이상이 백신접종 수용도가 높았는데, 연령대가 높아질수록 경력 또한 많아지므로 모두 동일한 결과라고 볼 수 있다. 연령이 적을수록 수용도가 감소하는 것을 예방하기 위하여 젊은 연령대를 대상으로 백신접종의 필요성에 대해 보다 적극적인 교육과 홍보가 필요할 것으로 생각된다.

한편 선호하는 백신이 없을수록 수용도가 높았는데, 이 부분은 백신의 안전성과 관련이 있으리라 생각된다. 본 연구 대상자의 평균 이상이 선호하는 COVID-19 백신이 있다고 응답하였으며, 대다수가 Pfizer 백신을 선호한다고 응답하였다. Merkley와 Loewen[22]의 연구에 따르면 사람들은 보고된 효능과 안전성 수준이 더 높은 백신을 접종하기를 원하며, 모든 백신이 안전하고 효과적이라고 간주됨에도 불구하고 Pfizer 백신과 Moderna 백신이 제공될 경우, Astrazeneca 백신과 Johnson & Johnson 백신접종을 훨씬 더 꺼리는 것으로 나타났다. 또한 이 백신들에 대한 주저는 매우 드물지만 심각한 부작용과의 연관성에 대한 논란이 제기되면서 접종 과정에서 더욱 커지는 것으로 나타났는데, 이는 본 연구에서도 동일하게 나타났다. 연구의 설문조사를 시행할 당시 Astrazeneca 백신접종 후 혈소판 감소증, 길랭-바레 증후군과 함께 혈전증이 최대 10,000명 중 1명에게 영향을 미칠 수 있음이 밝혀졌고[23], 국내에서도 이러한 이상반응 사례가 보고되었기에 선호하는 백신이 있을 수밖에 없는 상황이었다. 따라서 좀 더 많은 임상 결과를 가지고 안정성이 평가된 백신을 접종하기 위해 다른 사람들이 백신을 접종할 때까지 기다리는 것을 선호하였고[24], 본 연구에서도 백신접종 후 추천 의향을 묻는 문항에 20.9%가 ‘백신접종 연기를 권유할 것이다’라고 답했으며, 백신접종을 하지 않은 사람들의 55.2%가 ‘COVID-19 백신 종류에 따라 접종시기를 조절할 것이다’라고 답했다. 그러므로 백신의 개발, 승인 및 유통 과정, 효과, 이상반응, 그에 따른 정부의 대처 등 COVID-19 백신 전반에 걸쳐 투명성과 과학적 표준 준수를 강조하며 신뢰를 얻어야 할 것이다.

마지막으로 학사학위 이하 소지자에 비해 석사학위 이상 소지자에서 백신접종 수용도가 높았는데, 이 또한 선행연구 결과와 일치한다[14,15].

본 연구의 제한점은 다음과 같다. 첫째, 국가 정책 및 우선순위에 따라 고위험 의료기관 종사자, COVID-19 환자 치료병원 종사자로 1분기 예방접종 대상자인 간호사를 연구 대상자로 편의 표출하였기에 연구 결과를 일반화하는데 제한점이 있다. 둘째, 인터넷 사이트 이용자를 대상으로 하였으므로 대상자의 중복 가능성과 대리 응답으로 인한 정보편견 가능성을 배제할 수는 없다. 셋째, 수용도 관련 연구는 백신접종 전에 수용도와 수용도 관련 요인을 예측하는 것이 일반적이나, 본 연구는 COVID-19 백신접종 중에 시행한 연구로 선행연구와 직접적으로 수치를 비교하기에 어려움이 있다. 넷째, COVID-19 백신접종 시작 단계에서 연구를 시행하였기에 시간적 경과에 따라 변화되는 수용도에 대한 후속 연구를 제언한다.

본 연구의 장점은 다음과 같다. 첫째, COVID-19 백신접종 수용도에 대한 연구가 미흡한 국내 상황에서 비록 COVID-19 백신접종 시작 단계이지만 실제 접종여부를 파악하며, 수용도 수준과 수용도 관련 요인을 분석한 초기 연구라는데 의의가 있다. 둘째, 아동용 백신에 대한 기존의 연구도구를 저자의 승인을 받아 COVID-19 백신과 관련된 수용도를 파악할 수 있도록 도구를 수정, 보완하였고 전문가 그룹에게 내용타당도 검증을 실시하여 타당성을 높였다. 셋째, 수용도 관련 요인으로 나이, 경력, 선호 백신 여부, 학력을 확인하였으며 백신접종 수용도를 향상시키기 위한 교육 프로그램 개발의 기초자료가 된다는 점에서, 그리고 정책 방향을 제시한다는 점에서 의의가 있다.

## 결론

대상자들은 COVID-19 백신이 중요하고, 접종을 해야 한다고 생각하지만 백신의 안전성에 대한 수용도가 가장 낮았다. 수용도 관련 요인으로 나이와 경력이 많을수록, 선호하는 백신이 없을수록, 학력이 높을수록 COVID-19 백신접종 수용도가 높았다. 따라서 개인, 사회 문화적 차원에서 젊은 연령대를 대상으로 백신접종의 필요성과 가치에 관하여 보다 적극적인 교육이 요구되며, 국가적 차원에서 선호하는 백신에 대한 충분한 공급이 필요하다. 본 연구는 COVID-19 백신접종 시작 단계에서 실시된 연구로서, 세계적 대유행이 장기화됨에 따라 ‘위드 코로나(With COVID-19)’로 전환된 상황에 맞추어 백신접종 수용도에 대한 이해 변화를 평가하기 위한 비교 연구를 제언한다. 또한 본 연구에서 COVID-19 백신접종 수용도가 낮았던 젊은 연령대를 대상으로 백신접종의 필요성과 가치에 대한 홍보와 보건교육을 확대하여 수용도를 높이는 중재연구를 제언한다.

## Figures and Tables

**Table 1.** Scores for the Acceptance of COVID-19 Vaccination (N = 368)

Categories	Questions^†^	M ± SD
Safety		2.80 ± 0.83
	1. The COVID-19 vaccine is safe	3.95 ± 1.30
	2. The COVID-19 vaccine can cause severe adverse reactions (e.g., anaphylaxis)[RC]	2.58 ± 1.27
	3. The COVID-19 vaccine can cause mild adverse reactions (e.g. myalgia, fever, etc.)[RC]	1.56 ± 1.12
	4. COVID-19 infection may occur after COVID-19 vaccination [RC]	3.12 ± 1.54
Effectiveness and necessity		4.63 ± 0.86
	5. The COVID-19 vaccine is effective in preventing COVID-19 infection	4.98 ± 1.28
	6. Immunity acquisition by COVID-19 vaccination is similar to that caused by COVID-19 infection [RC]	3.62 ± 1.37
	7. The COVID-19 vaccine can prevent serious complications caused by COVID-19 infection	4.90 ± 1.46
	8. The COVID-19 vaccine has more benefits over risk	5.03 ± 1.50
Selection and scheduling		4.85 ± 1.24
	9. The start time of COVID-19 vaccination in Korea is appropriate	3.88 ± 1.70
	10. It is reasonable to vaccinate healthcare workers with the COVID-19 vaccine first	5.62 ± 1.50
	11. It is reasonable to vaccinate the older adults with the COVID-19 vaccine first	5.13 ± 1.58
	12. It is reasonable to vaccinate people with chronic diseases with COIVD-19 vaccine first	4.78 ± 1.73
Values and effect		4.97 ± 1.21
	13. COVID-19 vaccination can protect family and friends from COVID-19	5.32 ± 1.33
	14. COVID-19 vaccination can protect patients and other healthcare workers from COVID-19 infection	5.43 ± 1.36
	15. The COVID-19 vaccine can return to its previous routine	4.64 ± 1.62
	16. COVID-19 vaccination is unpleasant to me [RC]	4.49 ± 1.73
Legitimacy and authority		4.10 ± 1.02
	17. It is justifiable for the government to require healthcare workers to be vaccinated against COVID-19	4.62 ± 1.79
	18. The COVID-19 vaccination of healthcare workers should be voluntary [RC]	2.13 ± 1.30
	19. Healthcare workers must comply with the government's guidelines for COVID-19	5.52 ± 1.37
Total		4.28 ± 0.80

COVID-19 = Coronavirus disease 2019; M = Mean; SD = Standard deviation; RC = Reverse-coded items.

† The response range consisted of 1 to 7 points.

**Table 2.** Differences in the Acceptance of COVID-19 Vaccination according to Participants’ Demographic and Workplace Characteristics (N = 368)

Variables	n (%)	Acceptance of COVID-19 vaccination	
		M ± SD	t/F (*p*)
Gender			
Men	6 (1.6)	4.11 ± 0.64	-0.54 (.590)
Women	362 (98.4)	4.28 ± 0.79	
Age (yr)			
≤ 29^a^	167 (45.3)	4.06 ± 0.76	24.98 (< .001)
30-39^b^	153 (41.6)	4.31 ± 0.77	a < b < c
≥ 40^c^	47 (12.8)	4.93 ± 0.61	
No response	1 (0.3)		
Total		31.92 ± 6.65	
Marital status			
Unmarried	223 (60.6)	4.14 ± 0.80	-4.13 (< .001)
Married	145 (39.4)	4.48 ± 0.39	
Academic degree			
≤ Bachelor’s	270 (73.4)	4.15 ± 0.74	-5.33 (< .001)
≥ Master’s	98 (26.6)	4.63 ± 0.82	
Chronic disease			
Yes	27 (7.3)	4.42 ± 0.69	0.95 (.343)
No	341 (92.7)	4.27 ± 0.80	
Total clinical career (yr)			
≤ 5^a^	143 (38.9)	4.08 ± 0.73	20.38 (< .001)
6-10^b^	123 (33.4)	4.18 ± 0.81	a, b < c
≥ 11^c^	102 (27.7)	4.68 ± 0.71	
Total		8.27 ± 6.71	
Working hospital			
Tertiary general hospital	265 (72.0)	4.30 ± 0.83	0.36 (.699)^†^
General hospital	79 (21.5)	4.23 ± 0.66	
Hospital	24 (6.5)	4.21 ± 0.74	
The number of hospital beds			
≤ 299^a^	41 (11.2)	4.26 ± 0.62	1.49 (.230)^†^
300-999^b^	113 (30.7)	4.38 ± 0.71	
≥ 1000^c^	214 (58.1)	4.23 ± 0.85	
Work unit			
COVID-19 high-risk units	258 (70.1)	4.20 ± 0.78	2.88 (.004)
General ward	161 (43.8)		
Intensive care unit	62 (16.8)		
Emergency room	29 (7.9)		
COVID-19 care unit	6 (1.6)		
COVID-19 low-risk units	110 (29.9)	4.46 ± 0.79	
Outpatient	32 (8.7)		
Operating room	19 (5.2)		
Others	59 (16.0)		

COVID-19 = Coronavirus disease 2019; M = Mean; SD = Standard deviation.

† Welch's ANOVA.

**Table 3.** Differences in the Acceptance of COVID-19 Vaccination according to Participants’ COVID-19 Experience-related Characteristics (N = 368)

Variables	n (%)	Acceptance of COVID-19 vaccination	
		M ± SD	t/F (*p*)
COVID-19 exposure experience within 1 year			
Yes	94 (25.5)	4.24	-0.65 (.518)
No	274 (74.5)	4.30	
Type of COVID-19 exposure (n = 94)^†^			
Confirmed case	7 (7.4)		
Self-quarantine	34 (36.2)		
Active monitoring	33 (35.1)		
Only needed education	43 (45.7)		
Experience of caring a confirmed or close contact patient			
Yes	132 (35.9)	4.28 ± 0.74	-0.00 (.998)
No	236 (64.1)	4.28 ± 0.82	
Type of confirmed or close contact patients (n = 132)^†^			
Confirmed case	60 (45.5)		
Self-quarantine	61 (46.2)		
Active monitoring	39 (29.5)		
Only needed education	42 (31.8)		
No response	2 (1.5)		

COVID-19 = Coronavirus disease 2019; M = Mean; SD = Standard deviation.

† Multiple responses.

**Table 4.** Differences in Acceptance of COVID-19 Vaccination according to Vaccination related Characteristics of Participants (N=368)

Variables	n (%)	Acceptance of COVID-19 vaccination	
		M ± SD	t/F (*p*)
Influenza vaccination at last year			
Yes	329 (92.1)	4.28 ± 0.79	-0.26 (.799)
No	29 (7.9)	4.31 ± 0.80	
COVID-19 vaccination status			
Yes	330 (89.7)	4.30 ± 0.80	0.91 (.364)
No	38 (10.3)	4.17 ± 0.70	
Type of COVID-19 vaccine received (n = 330)			
Astrazeneca first	282 (85.5)		
Astrazeneca second	1 (0.3)		
Pfizer first	2 (0.6)		
Pfizer second	29 (8.8)		
No response	16 (4.8)		
Preferred COVID-19 vaccine			
Yes	226 (61.4)	4.17 ± 0.79	-3.49 (.001)
No	142 (38.6)	4.46 ± 0.76	
Type of preferred COVID-19 vaccine (n = 226)			
Pfizer	203 (89.4)	4.17 ± 0.77	-0.33 (.741)
Others	23 (10.6)	4.22 ± 0.92	
Information received prior to COVID-19 vaccination (n = 330)^†^			
Injection method	271 (82.1)		
Yes		4.33 ± 0.82	1.71 (.088)
No		4.10 ± 0.78	
Interval	284 (86.1)		
Yes		4.31 ± 0.83	0.92 (.360)
No		4.17 ± 0.66	
Effect	229 (69.4)		
Yes		4.36 ± 0.86	2.41 (.017)
No		4.13 ± 0.68	
Adverse reactions	299 (90.6)		
Yes		4.31 ± 0.83	2.54 (.019)
No		3.99 ± 0.48	
How to deal with adverse reactions	276 (83.6)		
Yes		4.34 ± 0.82	2.77 (.006)
No		3.06 ± 0.71	
General precautions	274 (83.0)		
Yes		4.37 ± 0.80	4.49 (< .001)
No		3.78 ± 0.73	
If you were vaccinated, would you recommend COVID-19 vaccination to others? (n = 330)			
Strongly recommend^a^	25 (7.6)	5.23 ± 0.46	88.70 (< .001) ‡
Encourage to follow the government's schedule^b^	197 (59.6)	4.53 ± 0.64	a > b > c > d
Postpone^c^	69 (20.9)	3.66 ± 0.53	
Recommend not to be vaccinated^d^	22 (6.7)	3.14 ± 0.84	
If not vaccinated, future plans (n = 38)			
To be vaccinated^a^	9 (23.7)	4.80 ± 0.45	9.58 (< .001)
Depending on the type of COVID-19 vaccine^b^	21 (55.2)	4.12 ± 0.65	a > b, c
Not to be vaccinated^c^	8 (21.1)	3.58 ± 0.47	

COVID-19 = Coronavirus disease 2019; M = Mean; SD = Standard deviation.

^†^Multiple responses; ^‡^Welch's ANOVA.

**Table 5.** Factors Related to Acceptance of COVID-19 Vaccination: Multiple Linear Regression (Variable Selection) (N = 368)

Variables	B	SE	*β*	t	*p*
(Constant)	4.24	0.07		61.65	< .001
Age (yr) (ref. group: ≤ 29)					
≥ 40	0.38	0.14	0.16	2.68	.008
Total clinical career (yr) (ref. group: ≤ 5)					
≥ 11	0.28	0.11	0.16	2.58	.010
Preferred COVID-19 vaccine (ref. group: No)					
Yes	-0.23	0.08	-0.14	-2.94	.004
Academic degree (ref. group: ≤ bachelor)					
≥ Master	0.21	0.10	0.12	2.05	.042
R^2^ = .16, Adjusted R^2^ = .15, F (*p*) = 16.83 (< .001)					

COVID-19 = Coronavirus disease 2019; ref. = Reference.

## Data Availability

Please contact the corresponding author for data availability.
